# Coarse-graining analysis for non-invasive, label-free, and quantitative evaluation of undulating motion in cultured oral mucosa epithelial cell sheets

**DOI:** 10.1016/j.reth.2026.101169

**Published:** 2026-09-02

**Authors:** Ren Minuma, Witsanu Yortchan, Ryota Kobayashi, Kenji Izumi, Yusuke Iida

**Affiliations:** a Graduate School of Science and Technology, Niigata University, 8050, Ikarashi 2, Nishi-ku, Niigata City, Niigata, 950-2181, Japan; b Division of Biomimetics, Faculty of Dentistry & Graduate School of Medical and Dental Sciences, Niigata University, 2-5274, Gakkocho-dori, Chuo-ku, Niigata City, Niigata, 951-8514, Japan; c Division of Pediatric Dentistry, Department of Preventive Dentistry, Faculty of Dentistry, Naresuan University, 99 Moo 9, Tha Pho Sub-district, Muang District, Phitsanulok, 65000, Thailand; d Division of Oral and Maxillofacial Surgery, Faculty of Dentistry & Graduate School of Medical and Dental Sciences, Niigata University, 2-5274, Gakkocho-dori, Chuo-ku, Niigata City, Niigata, 951-8514, Japan; e Faculty of Engineering, Niigata University, 8050, Ikarashi 2, Nishi-ku, Niigata City, Niigata, 950-2181, Japan

**Keywords:** Cultured oral mucosa epithelial cell sheet, Oral keratinocyte, Cell motility, Coarse-graining analysis, Quality control, Regenerative medicine

## Abstract

**Background:**

Cultured oral mucosa epithelial cell sheets (COMECS) represent a promising therapeutic approach for epithelial repair, such as corneal reconstruction or esophageal mucosa defects; however, current quality assessment methods are largely invasive and destructive. Although optical flow (OF)-based analysis has shown promise in evaluating keratinocyte motility, its sensitivity decreases under confluent conditions. A novel coarse-graining (CG) analysis combined with OF is introduced as a candidate non-invasive, label-free imaging parameter to quantitatively evaluate the characteristic undulating motion patterns (local directional synchrony of cell motion) of mature COMECS and to provide a potential basis for future quality assessment.

**Methods:**

COMECS were manufactured from passage 2 (p2) and passage 5 (p5) oral keratinocytes using standard (cultured in serum-containing medium) and sub-standard (cultured in serum-free medium) protocols. Time-lapse microscopy was conducted at multiple time points (48-, 96-, and 192-h post manufacture). The OF analysis-derived microscopic velocity fields were subjected to CG analysis to generate macroscopic velocity fields. The undulating index (UI) and mean undulating index (MUI) were calculated to quantify the directional synchrony of cellular motion. Western blotting was used to evaluate p63 and PITX1 protein expression levels to correlate the undulating motion patterns with biological markers of keratinocyte undifferentiation and oral epithelial identity.

**Results:**

OF-based CG analysis successfully differentiated undulating motion patterns between the standard and sub-standard COMECS, with average MUI values higher in the standard COMECS (*p* < 0.05). Time-course analysis of p2 COMECS indicated a substantially lower average MUI at 48 h than at 96 h and 192 h, suggesting time-dependent changes in collective motion behavior. A passage-dependent comparison showed apparent temporal patterns between p2 and p5 COMECS, with p5 sheets exhibiting earlier peak undulation but reduced sustainability. The p63 and PITX1 expression levels were elevated in standard COMECS, and were associated with enhanced undulating motion patterns.

**Conclusion:**

This novel OF-based, CG analysis enabled non-invasive, label-free, and quantitative assessment of COMECS quality using undulating motion pattern evaluation, demonstrating both applicability and feasibility for regenerative medicine quality control applications.

## Highlights

• A novel coarse-graining analysis quantified undulating motion in confluent cell sheets.
• OF-based CG analysis distinguished motion patterns between standard and sub-standard COMECS.
• Undulating motion was associated with p63–PITX1 axis and keratinocyte stemness.
• Time-course analysis indicated maturation-dependent changes in cell-cell adhesion.
• Passage-dependent patterns may reflect cell sheet handling period and stability.

## 1 Introduction

Cultured autologous oral mucosa epithelial cell sheets have emerged as a promising therapeutic approach for treating various epithelial defects, particularly corneal and esophageal reconstruction [1,2]. Despite considerable clinical progress, ensuring the quality of cultured oral mucosa epithelial cell sheets (COMECS) remains challenging. Current evaluation methods, such as cell counting, viability assays, keratinocyte marker analysis, immunostaining, and colony-forming assays, are largely invasive and destructive [2,3]. In addition, current quality assurance practices for regenerative medicine products rely almost exclusively on destructive testing and sampling inspection, making comprehensive batch-wide evaluation impossible [4,5]. As a result, clinicians must assess surrogate sheets rather than actual transplantable COMECS, which remains a major limitation to reliable quality control [6]. Recent advances in imaging and deep learning offer non-invasive alternatives. For example, Hirose et al. introduced DeepACT, a deep-learning-based automated cell tracking system that integrates cascading cell detection with Kalman filter algorithms to monitor individual keratinocyte movements within colonies in real time [7], providing a promising framework for non-destructive imaging-based evaluation in regenerative medicine.

In addition to individual cell tracking systems, optical flow (OF) analysis has been shown to enable non-invasive measurement of oral keratinocyte migration/displacement, indicated as motion speed (MS), which reflects cellular proliferative and regenerative capacity [8,9]. Therefore, OF-based approaches, which are widely applicable algorithms, have the potential to visualize and quantitatively evaluate keratinocyte motion behavior and offer a useful tool for quality evaluation in cultured epithelial cells. Although the OF-based protocol, which enables non-invasive observation of cell motion by means of pixel-level cell tracking, is effective for measuring MS in sub-confluent conditions, its sensitivity is lower in highly confluent conditions [9,10].

Time-lapse movies of COMECS indicate a characteristic undulating motion pattern (local directional synchrony of cell motion), such that simple application of, such as measuring MS alone, is inadequate for quantitatively evaluating COMECS motility. Therefore, an alternative image analysis method is required to analyze cell motility in densely packed or over-confluent cultured cells, such as COMECS. Coarse-grained (CG) analysis, traditionally used in molecular dynamics simulations to simplify complex molecular movements and compress computational data [11], can elucidate macromolecular behavior at reduced resolution scales. CG can therefore simplify cell motion data by averaging local movements, facilitating the analysis of large-scale collective cell motion behavior. By applying CG principles to the velocity fields derived from OF analysis, a novel OF-based image analysis was created that incorporated CG analysis to evaluate COMECS motion patterns.

The primary aim of this study was to examine the applicability of the original video analysis as a candidate non-invasive imaging parameter for the quantitative evaluation of the unique collective cellular motion of COMECS, specifically the undulating motion pattern, by comparing standard (cultured in a serum-containing medium) and sub-standard (cultured in a serum-free medium containing 1.2 mM Ca^2+^) protocols. To examine the biological relevance of these motion patterns, the protein expression levels of p63, a marker of undifferentiated basal keratinocytes, and paired-like homeodomain transcription factor 1 (PITX-1), an oral-type epithelial differentiation marker, were compared together with the quantified undulating motion patterns. The second aim was to evaluate the feasibility of the proposed imaging algorithm for COMECS time-lapse videos. An undulating index (UI) and mean undulating index (MUI) were determined as quantitative metrics to visually differentiate undulating motion patterns. Considering its potential as a basis for future quality assessment in regenerative medicine, the discriminative capability of the average MUI was examined under two different conditions: time-course and passage-dependent. By quantitatively examining COMECS motility patterns, the present study identified "undulating motion" as a specific characteristic that may provide a potential basis for future quality assessment. This approach addresses the need for non-invasive, label-free, quantitative, and real-time evaluation of confluent epithelial cell sheets and may provide a potential basis for future quality assessment in regenerative medicine applications.

## 2 Methods

### 2.1 Procurement of oral mucosa samples

The protocol for obtaining human oral mucosa tissue samples was approved by the Internal Review Board of the Niigata University (2015–5018). Patients who underwent minor dento-alveolar surgery were provided with sufficient information regarding this study, and all participating individuals signed an informed consent form.

### 2.2 Primary oral mucosa keratinocyte culture

Primary oral mucosa keratinocyte cultures were established, and the cells were serially sub-cultured as previously described [9,10]. Briefly, after excess blood on the tissue specimen was removed using a scalpel, the specimen was transferred to a 0.025% trypsin/ethylenediaminetetraacetic acid solution (Thermo Fisher Scientific, Waltham, MA, USA) containing 1.5% antibiotic–antimycotic solution (Thermo Fisher Scientific) and soaked for approximately 16 h at room temperature (25 °C). Oral mucosa keratinocytes were mechanically dissociated from the underlying connective tissue in a 0.0125% defined trypsin inhibitor (Thermo Fisher Scientific), resuspended in EpiLife® supplemented with EpiLife Defined Growth Supplements (EDGS: Thermo Fisher Scientific), 0.06 mM Ca^2+^, gentamicin (5.0 μg mL^−1^; Thermo Fisher Scientific), and amphotericin B (0.375 μg mL^−1^; Thermo Fisher Scientific) (referred to as Low Ca^2+^, serum-free, EpiLife), plated as p0 cells at a density of 4.0–5.0 × 10^4^ cells·cm^−2^, and fed with the same culture medium every other day. When the p0 cells were approximately 80% confluent, they were sub-cultured and serially passaged. In this study, COMECSs composed of p2 and p5 cells were used.

### 2.3 Production of COMECS for time-lapse microscopy under different conditions

To generate COMECS comprising of p2 cells, p1 oral keratinocytes were plated at a density of 3.0–4.0 × 10^5^ cells in five 35-mm dishes (Eppendorf, Hamburg, Germany) with Low Ca^2+^, serum-free, EpiLife medium, and continued to culture until cells became 100% confluent, while being fed every other day. Two to three days were taken for full confluence. Once cells were completely confluent, one group of COMECS in a 35-mm dish was manufactured under a sub-standard protocol, referred to as a “sub-standard” COMECS, in which the Low Ca^2+^ EpiLife medium was switched to a serum-free EpiLife containing 1.2 mM Ca^2+^ (High Ca medium). COMECS grown in the other four dishes were manufactured under a standard protocol in which the culture medium was switched to a 10% serum (fetal bovine serum; Corning, NY, USA) containing EpiLife®, referred to as the “standard” COMECS. This manufacturing protocol resulted in the initiation of p2 COMECS production, and 24 h later, each culture medium was refreshed. The standard and sub-standard COMECS were subjected to time-lapse microscopy from 48 to 96 h, immediately after refreshing the culture medium, to assess their applicability, followed by protein sample extraction from the COMECS (N = 7).

Subsequently, time-course and passage-dependent studies were conducted. For the time-course study, p2 standard COMECS were independently prepared for each observation time point, and separate dishes were subjected to time-lapse microscopy at 48, 96, and 192 h (N = 6 per time point). Thus, the three time-point groups were analyzed as independent samples rather than repeated measurements of the same COMECS. For the passage-dependent study, p4 oral keratinocytes were serially cultured from p1 cells derived from the same individuals in low-Ca2+, serum-free EpiLife medium. The p5 standard COMECS were then grown and subjected to time-lapse microscopy in the same manner (N = 6).

### 2.4 Time-lapse imaging and image processing

Five locations in a 35-mm dish were randomly-chosen and the standard and sub-standard COMECS were imaged by time-lapse microscopy at 8-min intervals for 4 h, using a CFI Plan Fluor DL4× objective lens equipped on a BioStudio-T imaging platform (Nikon, Tokyo, Japan) placed in a 5% CO_2_ incubator (Supplementary Video 1). A total of 31 images were converted to video files using Cell Image Viewer2 (Nikon). The video files were analyzed using the original image algorithm based on OF, followed by CG analysis. First, to examine the applicability of the image analysis method, video files of the standard and sub-standard COMECS were compared. To examine the feasibility of the MUI, the video files were then compared in the three different culture periods of the p2 standard COMECS, as well as in the p2 and p5 standard COMECS.

### 2.5 Coarse-graining analysis

**Fig. 1:**
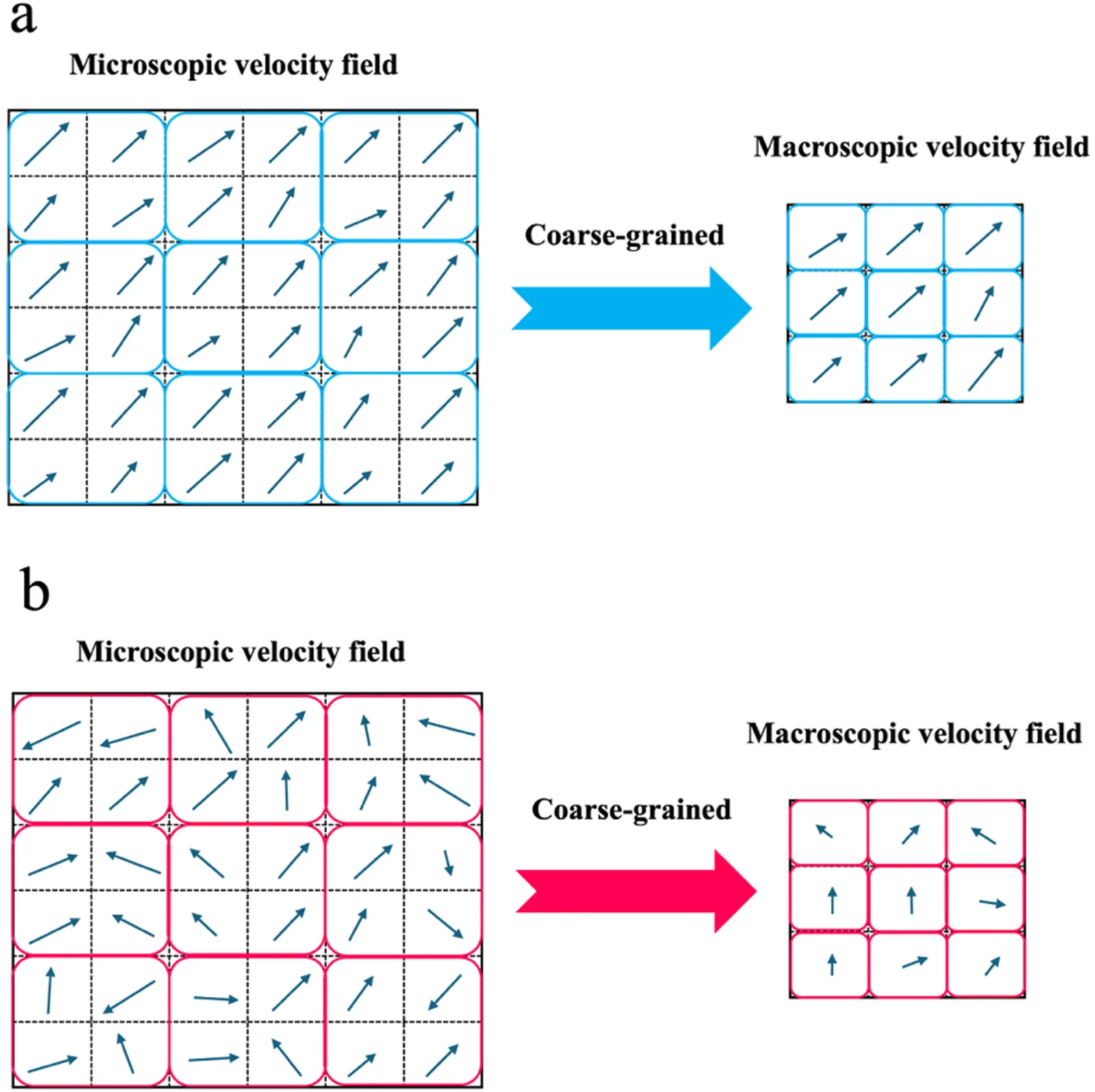
**Difference in macroscopic velocity due to local directional synchrony of the velocity field** (a) With the local directional synchrony, no marked changes in the absolute value of the macroscopic velocity after the artificial coarse-graining process were observed (b) Without the local directional synchrony, the absolute value of the macroscopic velocity is markedly decreased with the spatial coarse-graining process.

CG analysis was used to quantify the undulating motion of COMECS as a local, directional synchrony of cell motion. In CG analysis, the macroscopic velocity field was generated by applying spatial mosaic processing to the velocity field derived from OF. During this process, the magnitude of the macroscopic velocity was preserved better when the directional synchrony of the local vectors was higher (Fig. 1). Therefore, the undulation motion could be quantified by calculating the rate of change in velocity before and after mosaic processing.

#### 2.5.1 Derivation of velocity field in COMECS using optical flow (OF)

The correlation between the speed of cell migration and proliferative capacity using OF and the Farnebäck method has been reported [8,12]. In the current study, the two-dimensional velocity fields between consecutive frames of each time-lapse video was calculated using OF (Supplementary Video 2).

#### 2.5.2 Derivation of macroscopic relative velocity

First, the relative ratio of the decrease in the macroscopic velocity $V_{r}\left(s\right)$ was determined. First, the microscopic velocity field, V, derived from OF was expressed as:

(1) $$\begin{array}{c} V=\left(\begin{array}{ccc} v\left(1,1\right) & \cdots & v\left(N_{x},1\right)\\ \vdots & \ddots & \vdots \\ v\left(1,N_{y}\right) & \cdots & v\left(N_{x},N_{y}\right) \end{array}\right) \end{array}$$

where v(i,j) is the microscopic velocity calculated for each pixel by OF, i and j are positions in the image and integers that satisfy $1\leq i\leq N_{x},1\leq j\leq N_{y}$ when the number of pixels in the image is $N_{x}\times N_{y}$.

Due to the structural defects in the time-lapse microscope used in this study, inevitable micro-vibrations were macroscopically detected in the time-lapse video that disturbed the measurement of the real macroscopic velocity, compromising the CG analysis. Therefore, we applied a baseline correction to the microvibrations. The field of view was wide enough to contain sufficient cells for the analysis, and the motion due to vibration was simply estimated as the average of all velocities in the microscopic velocity field, v(i,j) (Supplementary Video 3). The microscopic velocity with the baseline correction, $v^{\prime }\left(i,j\right)$, was obtained as:

(2) $$\begin{array}{c} v^{\prime }\left(i,j\right)=v\left(i,j\right)-\frac{1}{N_{x}N_{y}}\sum_{1\leq i\leq N_{x},1\leq j\leq N_{y}\;}\;v\left(i,j\right) \end{array}$$

Next, the corrected microscopic velocity field, $V^{\prime }$**,** was divided into square regions of size, s×s pixels, and total number, $N_{mx}\times N_{my}$. Any region, $I_{i_{m},j_{m},s}$, contained a total of s×s microscopic velocities v and was described as:

(3) $$\begin{array}{c} I_{i_{m},j_{m},s}=\left\{\left(i,j\right)|\left(i_{m}-1\right)s+1\leq i\leq i_{m}s,\left(j_{m}-1\right)s+1\leq j\leq j_{m}s\;\right\} \end{array}$$

where $i_{m}$ and $j_{m}$ are the positions of the region and are integers satisfying $1\leq i_{m}\leq N_{mx},1\leq j_{m}\leq N_{my}$. $N_{mx}$ and $N_{my}$ are integers that satisfy $N_{mx}=\lfloor N_{x}/s\rfloor ,N_{my}=\lfloor N_{y}/s\rfloor$.

The macroscopic velocity field $v_{m}^{\prime }\left(i_{m},j_{m},s\right)$ in each segmented region $I_{i_{m},j_{m},s}$ was then derived by calculating the average $v^{\prime }\left(i,j\right)$ of the microscopic velocities in each segmented region, $I_{i_{m},j_{m},s}$. The velocity of position $\left(i_{m},j_{m}\right)$ in the macroscopic velocity field when coarse-grained on a spatial scale s was defined as $v_{m}^{\prime }\left(i_{m},j_{m},s\right)$, and was calculated as:

(4) $$\begin{array}{c} v_{m}^{\prime }\left(i_{m},j_{m},s\right)=\frac{1}{s^{2}}\sum_{i,j\in I_{i_{m},j_{m},s}}v_{i,j}^{\prime } \end{array}$$

The average absolute value of the velocity in the macroscopic velocity field, $\bar{V_{m}\left(s\right)}$, was then calculated from Equation (5):

(5) $$\begin{array}{c} \bar{V_{m}\left(s\right)}=\frac{1}{N_{mx}N_{my}}\sum_{1\leq i_{m}\leq N_{mx},1\leq j_{m}\leq N_{my}\;}\left|v_{m}^{\prime }\left(i_{m},j_{m},s\right)\right| \end{array}$$

Finally, the macroscopic relative velocity, $V_{r}\left(s\right)$, which was used as a quantitative parameter with spatial scale, s, was obtained as:

(6) $$\begin{array}{c} V_{r}\left(s\right)=\frac{\bar{V_{m}\left(s\right)}}{\bar{V_{m}\left(1\right)}} \end{array}$$

#### 2.5.3 Derivation of UI

The UI was determined by the rate of change of the macroscopic relative velocity $V_{r}\left(s\right)$ as the spatial scale, s, increased. This metric was used to quantify the local directional synchrony of the microscopic velocity field. In the current study, UI was calculated using the macroscopic relative velocities obtained at the spatial scales of 5, 10, 15, 20, and 25. Differences in the local directional synchrony appeared as the rate of change in $V_{r}\left(s\right)$ (Supplementary Fig. S1).

To derive UI, $V_{r}\left(s\right)$ was quantified by a linear least-squares fitting of each velocity field. For a comprehensive quantitation of the undulating motion of a time-lapse video, MUI was calculated as the average UI for all 30 frames in the video. Time-lapse imaging was conducted at five randomly-chosen locations within each COMECS. Therefore, the average MUI was calculated by averaging five MUI values, enabling a quantitative evaluation of the undulating motion per sample.

### 2.6 Western blot analysis

Western blotting was conducted to compare the protein expression levels of p63 and PITX1 with the quantified undulating motion patterns under standard and sub-standard conditions. After time-lapse microscopy, the p2 COMECS were washed three times with cold phosphate-buffered saline and then lysed with 200 μL of lysis buffer (Cell lysis buffer solution (10X) (Cell Signaling Technology, Danvers, MA, USA), double distilled water, and phenylmethanesulfonyl fluoride (Cell Signaling Technology)). Cell lysates were ultrasonicated and centrifuged at 14000 rpm for 10 min at 4 °C. The supernatant was collected and denatured at 70 °C for 10 min. Protein samples were loaded and separated onto polyacrylamide gels (Bolt 4–12% Bis-Tris Plus Mini Protein Gel, Thermo Fisher Scientific) and then transferred to polyvinylidene difluoride membranes (Thermo Fisher Scientific). The non-specific binding sites of all membranes were blocked using blocking buffer (StartingBlock Blocking buffer, Thermo Fisher Scientific) and then incubated overnight at 4 °C with the primary antibodies of p63α (D2K8X) (rabbit polyclonal, 1:1000, 4892S, Cell Signaling Technology), PITX-1 (rabbit polyclonal, 1:1000, 10873-1-AP, Proteintech, Rosement, IL, USA) and β-actin (rabbit polyclonal, 1:1000, 4970s, Cell Signaling Technology). After primary incubation, all membranes were washed with Tris-buffered saline containing 0.05% Tween 20 (TBST) and incubated with a horseradish peroxidase-conjugated secondary antibody (GE Healthcare UK Ltd., Little Chalfont, Buckinghamshire, UK) for 1.5 h at room temperature. Protein bands were visualized using western enhanced chemiluminescence substrate (Bio-Rad Laboratories, Hercules, CA, USA), and images were acquired using the CHEMIDOC XRS Plus + IMAGE LAB software (Bio-Rad Laboratories). β-actin expression was used as a loading control.

### 2.7 Statistical analysis

For the comparison between standard and sub-standard COMECS, the Wilcoxon signed-rank test was used. The same test was also used for the passage-dependent comparison between p2 and p5 standard COMECS derived from the same individuals. For the time-course study of p2 standard COMECS at 48, 96, and 192 h, the samples analyzed at each time point were independent and were not repeated measurements of the same COMECS. Because the sample size was small and normality could not be assumed, differences among the three groups were analyzed using the Kruskal–Wallis test. When the overall comparison was significant, Dunn's multiple-comparison test with adjustment for multiplicity was used for post hoc pairwise comparisons. A p-value of < 0.05 was considered statistically significant. Statistical analyses were performed using the SciPy library (version 1.13.1).

## 3 Results

### 3.1 Quantitative assessment of the undulating motion pattern of COMECS was comparable to visual inspection

**Fig. 2:**
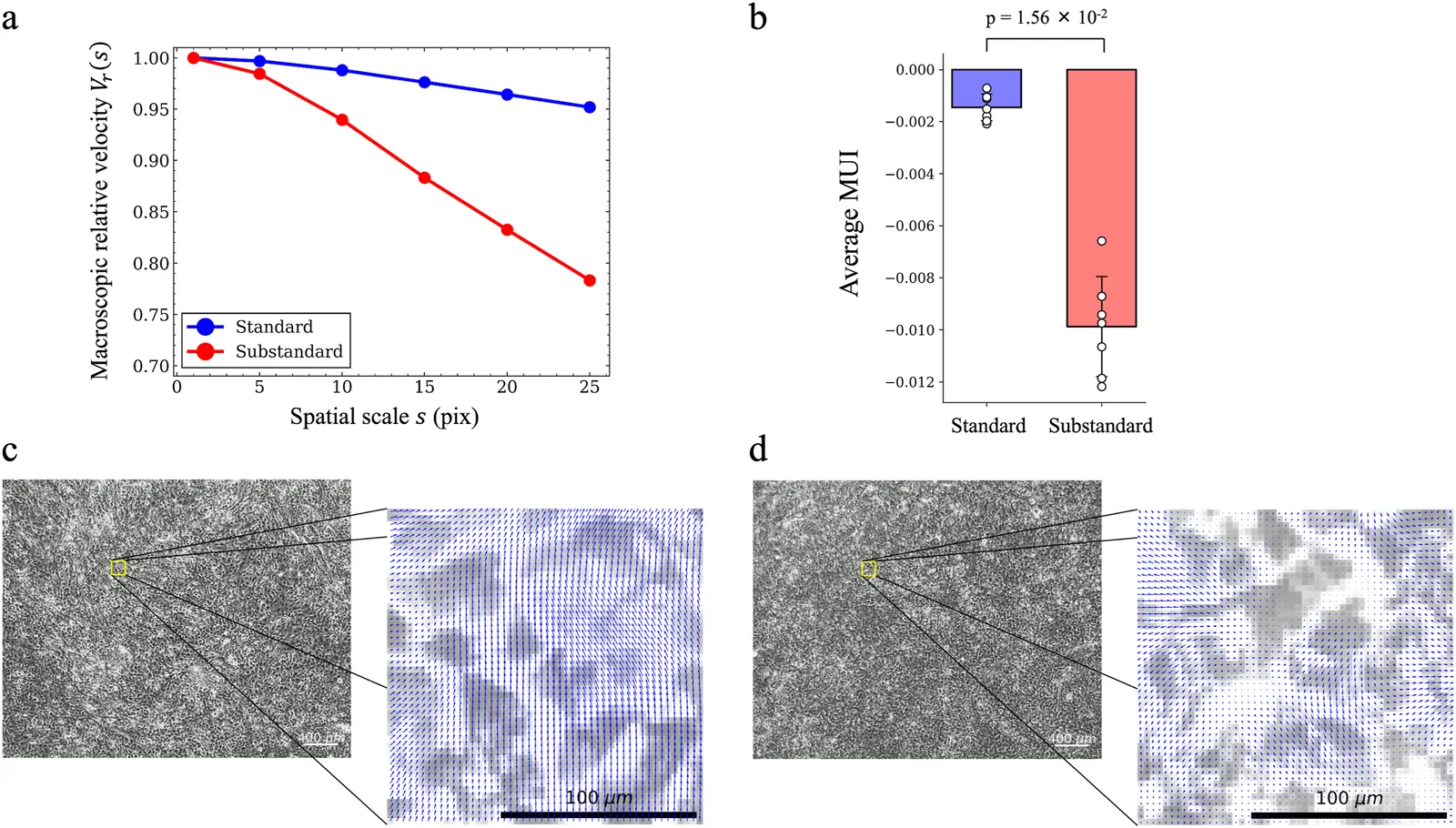
**The original algorithm of this study, based on optical flow (OF) followed by coarse-graining (CG) analysis, differentiated and quantified the passage 2 (p2) cultured oral mucosa epithelial cell sheet (COMECS) motion patterns between standard and sub-standard manufacturing protocols** (a) Representative data (macroscopic relative velocity, $V_{r}\left(s\right)$) indicate a distinct downward tendency between the p2 standard and sub-standard COMECS. The horizontal and vertical axes represent the spatial scale, s, and macroscopic relative velocity $V_{r}\left(s\right)$, respectively. The blue and red lines indicate the standard and sub-standard COMECS, respectively. (b) Average “mean undulating index” (MUI) (an index of cell undulating level obtained from 30 frames of time-lapse microscopic images) of the standard and sub-standard COMECS. Dots represent the average MUI of each sample (N = 7). Data are shown as mean ± standard deviation (SD). The p2 standard COMECS had a higher average MUI than p2 sub-standard COMECS (p<0.05; Wilcoxon signed-rank test). (c) A typical standard COMECS microscope image and visualization of the microscopic velocity field. The full microscope image consists of 1024 ×1280 pixels, and the figure shows a magnified view of a local region (50× 50 pixels). The velocity field is indicated by blue arrows in the magnified image. The magnified image reveals a high degree of local directional synchrony. (d) A typical substandard COMECS microscopy images and visualization of the microscopic velocity field. The full microscopy image consists of 1024 ×1280 pixels, and the figure shows a magnified view of a local region (50× 50 pixels). The velocity field is indicated by blue arrows in the magnified view. Each vector is scaled by a factor of 2.5 for visualization. Compared to the magnified view in (c), the local directional synchrony is lower.

First, the applicability of the algorithm to the original OF-based CG analysis was determined using standard and sub-standard COMECS (derived from the same individual oral keratinocytes) manufactured under each protocol. The standard COMECS clearly showed faster cell migration and greater undulating motion than the sub-standard COMECS under visual inspection (Fig. 2c and d, Supplementary Videos 1 and 2). When the algorithm was applied, the macroscopic relative velocity of the standard COMECS tended to decrease gradually as the CG spatial scale increased, whereas the decline rate in the sub-standard COMECS was steep in all 1020 frames (Fig. 2a). Therefore, the original algorithm was able to differentiate and quantify the undulating motion pattern between the standard and sub-standard COMECS, indicating the applicability of the algorithm using CG analysis to measure the unique, collective, cellular motion of COMECS using the MUI. The micro-vibrations found in 51 out of 1020 frames in the time-lapse videos caused an increase in the macroscopic velocity field in the sub-standard COMECS. This unavoidable defect compromised the CG image analysis and resulted in a reverse macroscopic relative velocity compared to the standard COMECS. Therefore, our baseline correction was applied to remove the false velocity vectors caused by micro-vibrations (Supplementary Fig. S2), resulting in the complete correction of the false velocity vectors in all frames (Supplementary Fig. S3). The consistent gradual decline in the macroscopic relative velocity in the standard COMECS was compared to the steep decline in the sub-standard COMECS and a baseline correction was applied to all CG analyses.

To confirm the applicability of the algorithm, we compared the downward trends observed in standard and sub-standard COMECS. Using seven samples, we plotted the mean values of five MUIs, which were obtained from one of five videos in each COMECS (a single MUI was calculated as the mean of 30 UIs from one video). The results showed that the average MUI of standard COMECS was higher than that of sub-standard COMECS, although both remained below zero (Fig. 2b). This indicates the ability of the algorithm to differentiate and quantify the characteristic undulating motion of COMECS in a manner consistent with visual inspection.

### 3.2 Changes in average MUI patterns in a time-course study of p2 standard COMECS

**Fig. 3:**
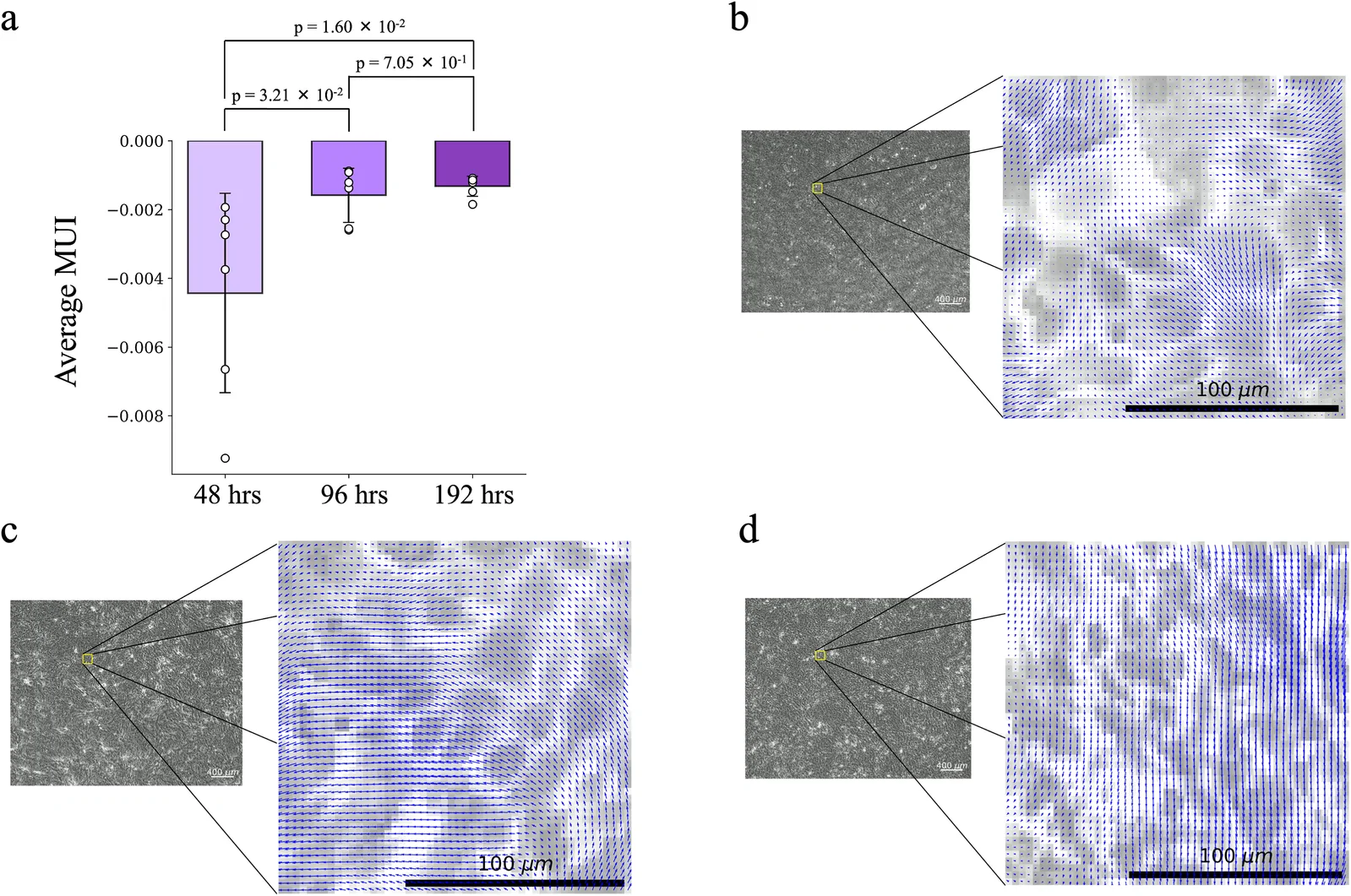
**Changes of average MUI over time in the same passage 2 (p2) standard cultured oral mucosa epithelial cell sheets (COMECS)** (a) Data are shown as mean ± standard deviation (SD). Independent dishes were analyzed at each time point (N = 6 per time point). The average MUI of p2 standard COMECS was lowest at 48 h and was significantly lower than those at 96 h and 192 h, whereas no significant difference was observed between 96 h and 192 h. Differences among the three groups were evaluated using the Kruskal–Wallis test (H = 9.14, p = 0.010), followed by Dunn's post hoc test for multiple comparisons (48 h vs 96 h, p = 3.21 × 10−2; 48 h vs 192 h, p = 1.60 × 10−2; 96 h vs 192 h, p = 7.05 × 10−1). The p-values shown in the figure are Holm-adjusted. (b) (c) (d) Microscopic images and visualizations of the microscopic velocity field for a typical p2 standard COMECS. The images shown are from 48 h (b), 96 h (c), and 192 h (d), respectively. The full microscopic images consist of 1024 × 1280 pixels, and the figures show enlarged views of a local region (50 × 50 pixels) within each image. The velocity field is indicated by blue arrows in the enlarged images. For visualization, the scale factor for each vector is 3.33x in (b) and 1.67x in (c) and (d). Comparing the enlarged images in (c) and (d) with (b) reveals that (b) exhibits lower local directional synchrony.

Temporal changes in the average MUI were examined using p2 standard COMECS at different time points after COMECS initiation. Independent cell sheets were analyzed at 48, 96, and 192 h after COMECS initiation (Fig. 3). The average MUI of standard COMECS at 48 h was substantially smaller than those at 96 h and 192 h, whereas the values at 96 h and 192 h were similar. These findings suggest that the undulating motion increased (became closer to the 0 level) between 48 h and 96 h after COMECS initiation and was then maintained through 192 h, indicating time-dependent changes in collective motion behavior.

### 3.3 Differences in passage-dependent average MUI of COMECS

**Fig. 4:**
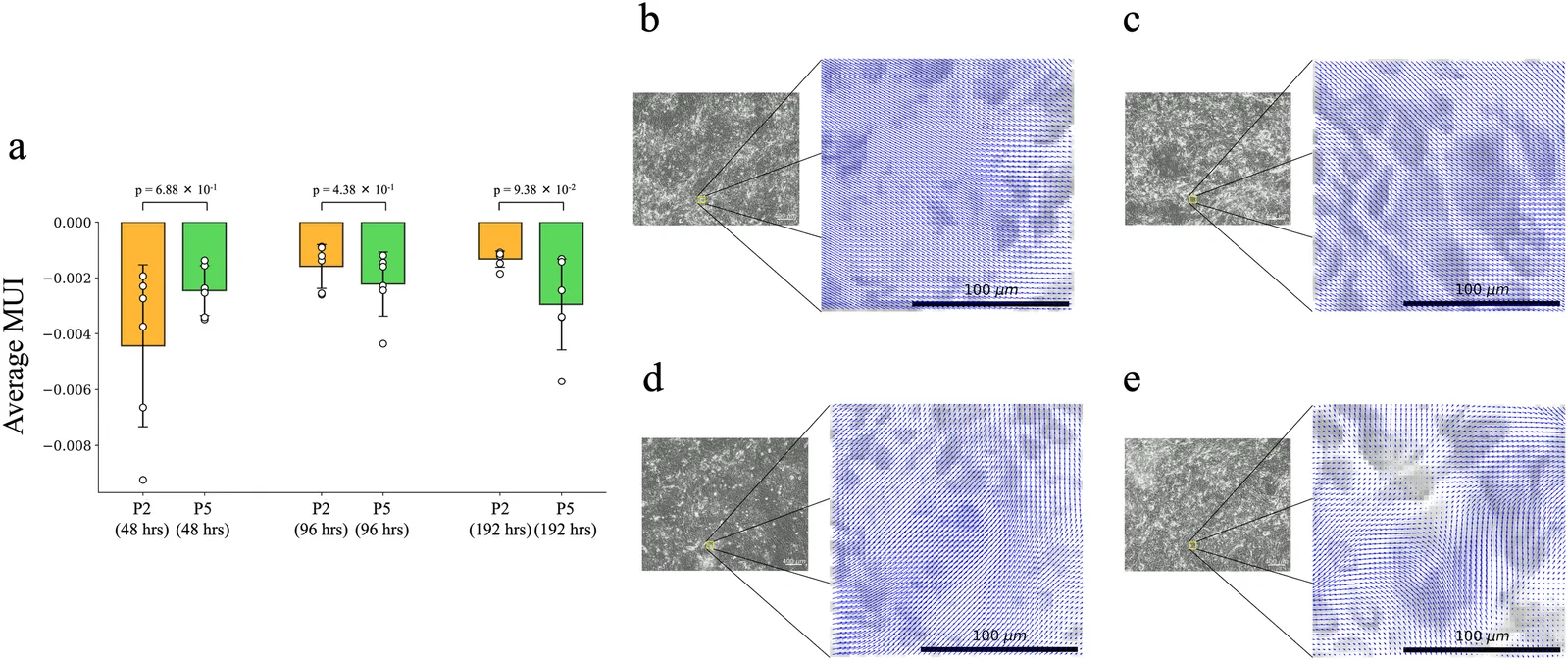
**Differences in average MUI of cultured oral mucosa epithelial cell sheets (COMECS) of different passages** (a) Data are shown as mean ± standard deviation (SD). At 192 h, the average MUI of p2 COMECS tended to be higher than that of p5 COMECS; however, no statistically significant differences were observed between the two groups at 48, 96, or 192 h (Wilcoxon signed-rank test, N = 6). (b) (c) Visualization of the microscopic velocity field for typical p2 and p5 standard COMECS on 48 h. (b) and (c) show p2 and p5, respectively. The entire microscopic image consists of 1024 × 1280 pixels, and the figures show enlarged views of a local region (50 × 50 pixels) within it. The velocity field is indicated by blue arrows in the enlarged views. Each vector is scaled by a factor of 2.0 for visualization. No differences in local directional synchrony were observed when comparing the enlarged views in (b) and (c). (d) (e) Visualization of the standard COMECS macroscopic velocity field for typical p2 and p5 cells on 192 h. (d) and (e) show p2 and p5 cells, respectively. The full microscopic image consists of 1024 × 1280 pixels, and the figures show enlarged views of a local region (50 × 50 pixels) within it. The velocity field is indicated by blue arrows in the enlarged views. For visualization, the scale factor for each vector is 1.67x in (d) and 2.0x in (e). Comparing the two figures, the local directional synchrony in (d) is higher than that in (e).

Differences in the average MUI of standard COMECS generated from cells from passage 2 (p2) and 5 (p5) were compared. Standard COMECS were analyzed at 48, 96, and 192 h after COMECS initiation (Fig. 4). No clear difference was observed between p2 and p5 at 48 h and 96 h. However, at 192 h, the average MUI of p2 standard COMECS tended to be higher than p5 standard COMECS, although no statistically significant differences were observed between groups.

### 3.4 Western blotting

**Fig. 5:**
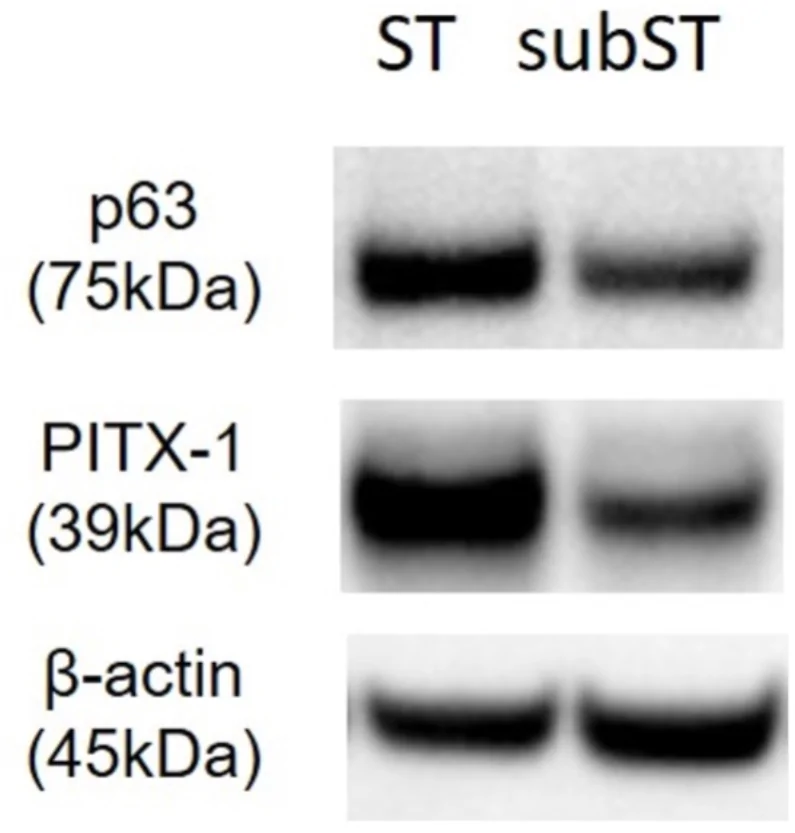
**Representative Western blot image of p63 and PITX-1 of the passage 2 (p2) standard (ST) and sub-standard (subST) cultured oral mucosa epithelial cell sheets (COMECS)** β-actin was used as a loading control.

PITX1 and p63 function as markers of oral-type epithelial differentiation and of basal or progenitor cells, respectively. The protein expression levels of p63 and PITX1 in the p2 standard COMECS were higher than those in the p2 sub-standard COMECS when examining the samples after time-lapse microscopy at 48–96 h (Fig. 5; Supplementary Fig. S4). Densitometric analysis of the three biological replicates, normalized to β-actin, supported the same overall trend, although these findings should be interpreted with appropriate caution given the limited sample size.

## 4 Discussion

Non-invasive, label-free, and real-time quality control is increasingly important for cell-based products in regenerative medicine because it enables repeated in-process evaluation without compromising the final therapeutic product [13,14]. Recent advances in image analysis, including deep-learning-based approaches, have demonstrated the potential of non-invasive quality assessment in various cell types. In particular, static image-based morphological analyses have shown promise for mesenchymal stem cells (MSCs) and induced pluripotent stem cells (iPSCs) [15,16], and similar applications are beginning to emerge for epithelial cells and keratinocytes [17,18]. However, time-lapse video analysis remains underused in this field, despite its potential to capture dynamic cellular behaviors that are not reflected by static morphology alone.

Although oral mucosa epithelial keratinocyte motility, particularly movement velocity, has been reported to reflect proliferative and differentiation capacity [9,10], those studies focused on proliferative-phase cells at relatively low confluence and were not designed to evaluate mature cultured oral mucosa epithelial cell sheets (COMECS). In the present study, we applied coarse-graining (CG) analysis to mature COMECS and quantitatively evaluated their characteristic undulating motion patterns.

CG analysis is based on local synchrony of cellular motion. When cells exhibit undulating movement, motion in one region influences neighboring cells and generates local directional synchrony; in the absence of such behavior, cells move more independently. On this basis, we analyzed microscopic velocity fields obtained by optical flow (OF) and applied artificial mosaic processing, termed “coarse-graining,” to quantify directional synchrony/coherence. Unlike deep-learning-based approaches such as DeepACT [7], this method is rule-based and does not require training datasets or human-dependent annotation. By combining OF with CG analysis, our approach enabled quantitative evaluation of collective cellular behavior in mature epithelial sheets.

Our results showed that OF-based CG analysis could distinguish the undulating motion patterns of COMECS produced under the standard protocol from those produced under the sub-standard protocol, supporting its applicability as a candidate non-invasive, label-free imaging parameter for evaluating motion patterns in COMECS. This comparison is particularly relevant because COMECS are clinically manufactured using autologous cells, typically with autologous serum supplementation. Notably, mean motion speed (MMS), which has been useful in previous studies of keratinocyte motility [[8], [9], [10]], also showed a significant difference between standard and sub-standard COMECS (Supplementary Fig. S5). However, because MMS quantifies only the magnitude of motion and not its directionality, it does not directly evaluate the local directional synchrony that defines the undulating motion pattern of COMECS. Thus, while MMS may provide supportive information on overall cell movement, CG analysis appears more suitable for evaluating the characteristic motion pattern of confluent COMECS.

The biological relevance of these motion patterns was supported by molecular analyses. Expression of p63, a marker of undifferentiated basal keratinocytes, was higher in standard COMECS than in sub-standard COMECS. This is consistent with previous reports showing that p63 expression in epithelial cell sheets is associated with favorable clinical outcomes [19,20], as well as with the established role of p63 in maintaining the undifferentiated keratinocyte state [21]. Because a higher proportion of undifferentiated cells in COMECS is associated with better post-transplantation outcomes, serum supplementation may positively influence wound healing, tissue regeneration, and graft success [22]. PITX1 expression was also correlated with p63 expression, consistent with the known role of p63 as an upstream regulator of PITX1 in oral keratinocytes. Given that PITX1 contributes to oral epithelial identity and promotes keratinocyte migration and proliferation [23,24], the undulating motion observed in standard COMECS may reflect, at least in part, preservation of the p63-PITX1 axis and oral epithelial characteristics. However, these molecular findings should be interpreted as biological correlates of cellular status rather than as direct evidence of the driving mechanism of undulating motion.

Time-course analysis of passage 2 (p2) standard COMECS showed that the average MUI was lower at 48 h than at 96 h and 192 h after COMECS initiation, while remaining high from 96 h to 192 h. In addition, although not shown in the data, confluent cells cultured in low-calcium, serum-free EpiLife medium immediately before COMECS production exhibited little or no collective directional motion, whereas this became apparent after serum addition. Together, these findings suggest time-dependent changes in the collective motion behavior of standard COMECS. Thus, the low average MUI at 48 h may reflect an earlier state of cell-sheet organization, whereas the higher MUI after 96 h may be associated with a more developed collective motion pattern. Although these changes may be related to the establishment of cell-cell adhesion and structural stability, neither was directly assessed in the present study. Further studies are needed to clarify the relationship between CG-derived parameters, mechanical strength, and the biological basis of these motion patterns. Nonetheless, these findings support the value of OF-based CG analysis as a candidate non-invasive and label-free imaging parameter for evaluating motion patterns in cell sheets.

Passage-dependent comparison between p2-and p5-derived standard COMECS also revealed apparent temporal patterns, although the differences did not reach statistical significance. The p5 standard COMECS appeared to attain synchronized motion earlier than the p2 standard COMECS, but this state was less sustained and declined at 96 h and 192 h after COMECS initiation. Compared with p2 standard COMECS, the period during which p5 standard COMECS were easy to handle appeared to be shorter, after which the sheets became more difficult to manipulate. These observations suggest that quantification of undulating cellular motion may allow non-invasive detection of temporal deterioration in cell-cell adhesion and sheet stability.

The mechanistic basis of enhanced motility in stem/progenitor keratinocytes remains to be clarified. Nanba et al. reported that highly motile epidermal stem cells exhibit elevated Rac1 activity and shortened actin filaments, thereby facilitating cellular movement [25]. High integrin α6 expression may also contribute to the anchoring required for efficient motility. In addition, molecules involved in cell-cell adhesion molecules and cytoskeletal regulators may play important roles in the generation and maintenance of the collective motion observed in COMECS. Further investigation is needed to determine how the undulating motion patterns identified here relate to these molecular mechanisms and to wound-healing capacity.

Overall, the present study demonstrated that video analysis of COMECS using CG methodology can serve as a candidate non-invasive imaging parameter for evaluating undulating motion patterns. OF-based CG analysis differentiated and quantified the undulating motion patterns of COMECS produced under standard and sub-standard manufacturing protocols, and time-course and passage-dependent analyses suggested that average MUI may reflect temporal changes relevant to cell-sheet handling and stability. Future studies should determine whether CG-derived parameters correlate with handling properties, mechanical strength, and clinically relevant regenerative outcomes, and whether this approach can be extended to other epithelial cell-sheet products. Such validation will be important to establish whether this methodology may provide a potential basis for future quality assessment in regenerative medicine.

## Ethics declaration

Written informed consent to take part in the study and to publish the article has been obtained from all participants or their legal representatives. The privacy rights of participants have been observed.

This study included organ or tissue donors. This study includes human biological material and consent was obtained by donors, or their next of kin or legal representatives, for use in this study and for publication of the article. The samples used in this research were not sourced from executed prisoners or prisoners of conscience.

This study was performed in compliance with relevant laws, regulatory frameworks and guidelines where the research took place. This study was approved by the Internal Review Board of the Niigata University. (Approval No. 2015–5018)

## Funding information

This work was supported by 10.13039/501100001691JSPS KAKENHI Grant Number 24K15822 to R.K. and the Terumo Life Science Foundation to Y.I.

## Declaration of competing interest

No competing financial interests exist for this work.

## Appendix A Supplementary data

Video 1

Video2
