# Supplementary material for: Coarse-graining analysis for non-invasive, label-free, and quantitative evaluation of undulating motion in cultured oral mucosa epithelial cell sheets
Source: Regen Ther. 2026 Sep 2;33:101169. doi: 10.1016/j.reth.2026.101169 (PMC13571252; doi:10.1016/j.reth.2026.101169)
Supplement: Supplementary file 1 [file mmc1.pdf]

Supplementary Materials

Supplementary Video 1

Representative video of standard (left) and substandard COMECS

Supplementary Video 2

Representative video, having vectors after OF application, standard (left) and substandard COMECS

Supplementary Video 3

Representative video of standard COMECS before (left) and after (right) baseline correction.

Supplementary Figures

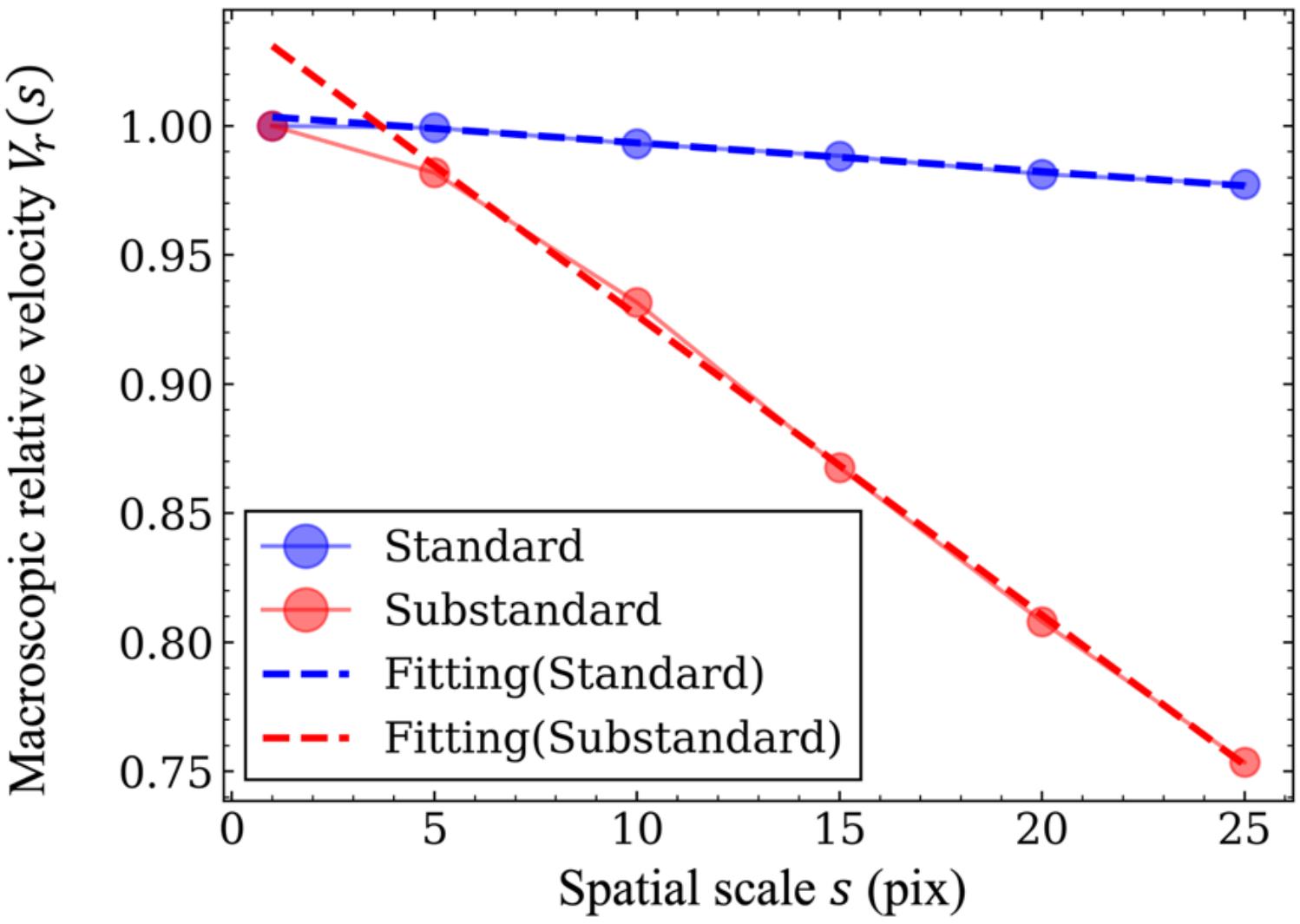

Supplementary Figure S1 Dependence of $V_r(s)$ on spatial scale, s

The blue solid line represents the spatially synchronous case and the solid red line represents the non-synchronous case. Both the blue and red dotted lines show the results of the least-squares fitment

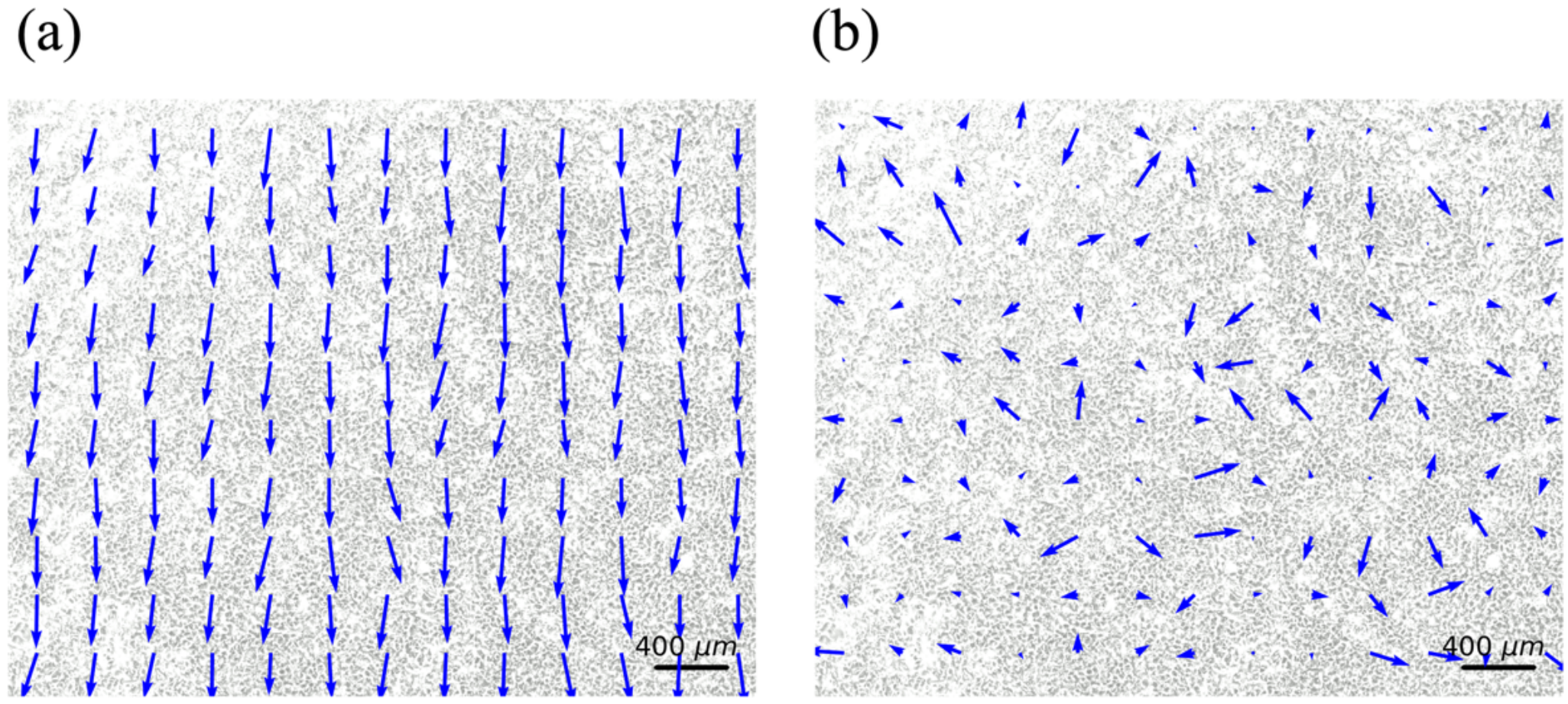

Supplementary Figure S2 Examples of macroscopic velocity fields before (a) and after (b) baseline correction

(a) Coarse-grained macroscopic velocity field visualizes the average directional synchrony of cell motion within relatively large local regions. To visualize relatively large regions, the spatial scale, s, which determines the coarse-graining region size, was set to 100. This velocity field is affected by micro-vibrations, and the vectors exhibiting macroscopic velocity across the entire field of view show directional alignment. Each vector is scaled by a factor of 100 for visualization.

(b) Coarse-grained macroscopic velocity field visualizes the average directional synchrony of cell motion within relatively large local regions. To visualize relatively large regions, the spatial scale, s, which determines the coarse-graining region size, was set to 100. After applying baseline correction to the velocity field shown in (a), the vectors

exhibiting macroscopic velocity in the field of view display highly heterogeneous orientations. Each vector is scaled by a factor of 300 for visualization.

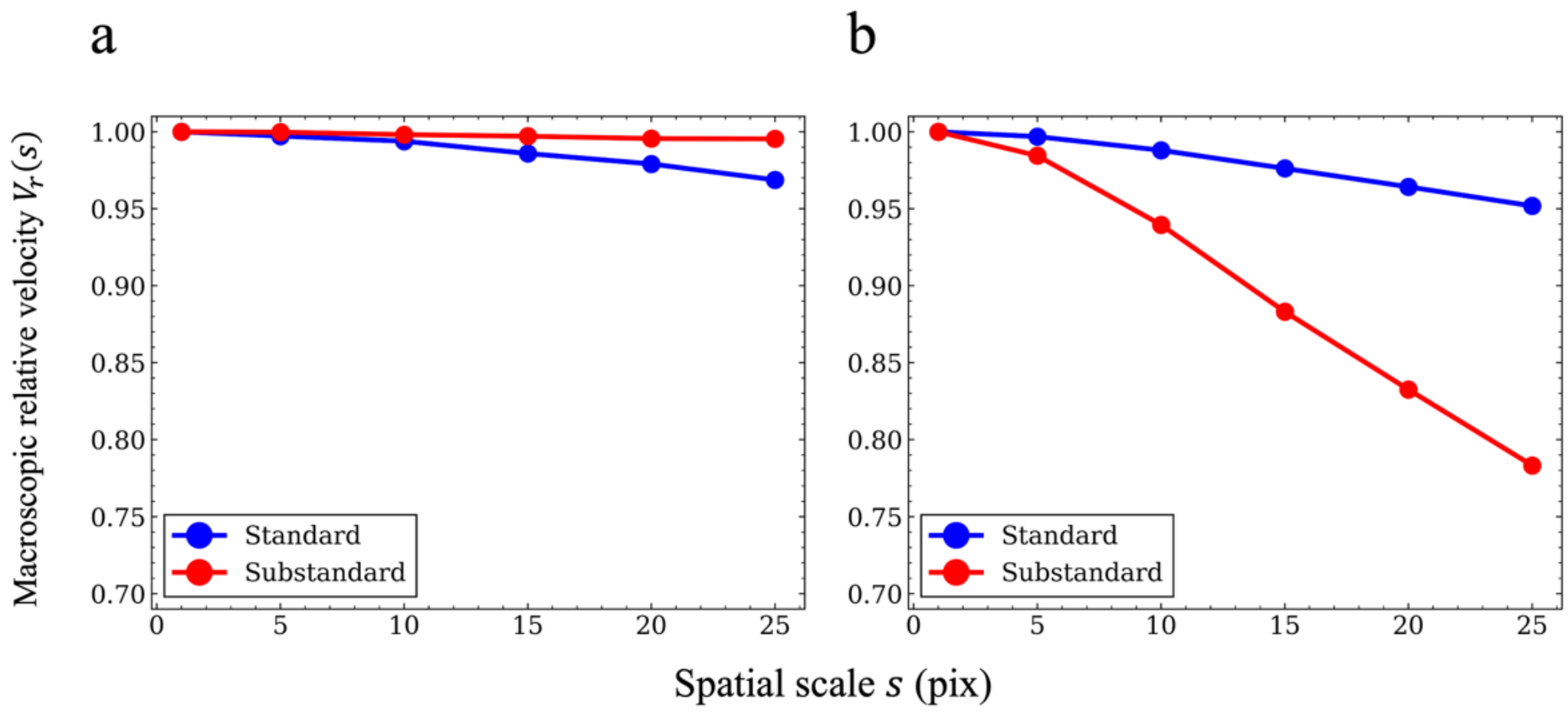

Supplementary Figure S3 Examples of coarse-graining (CG) analysis results before and after baseline correction

(a) Data (macroscopic relative velocity, $V_r(s)$) before baseline correction. Data tended to decrease with a more gradual rate of change in sub-standard COMECS, which was different from visual inspection

(b) Data (macroscopic relative velocity, $V_r(s)$) after the baseline correction. Compared to the data before baseline correction shown in (a), the rate of change of substandard COMECS was substantially reduced and concurred with visual inspection

(a)

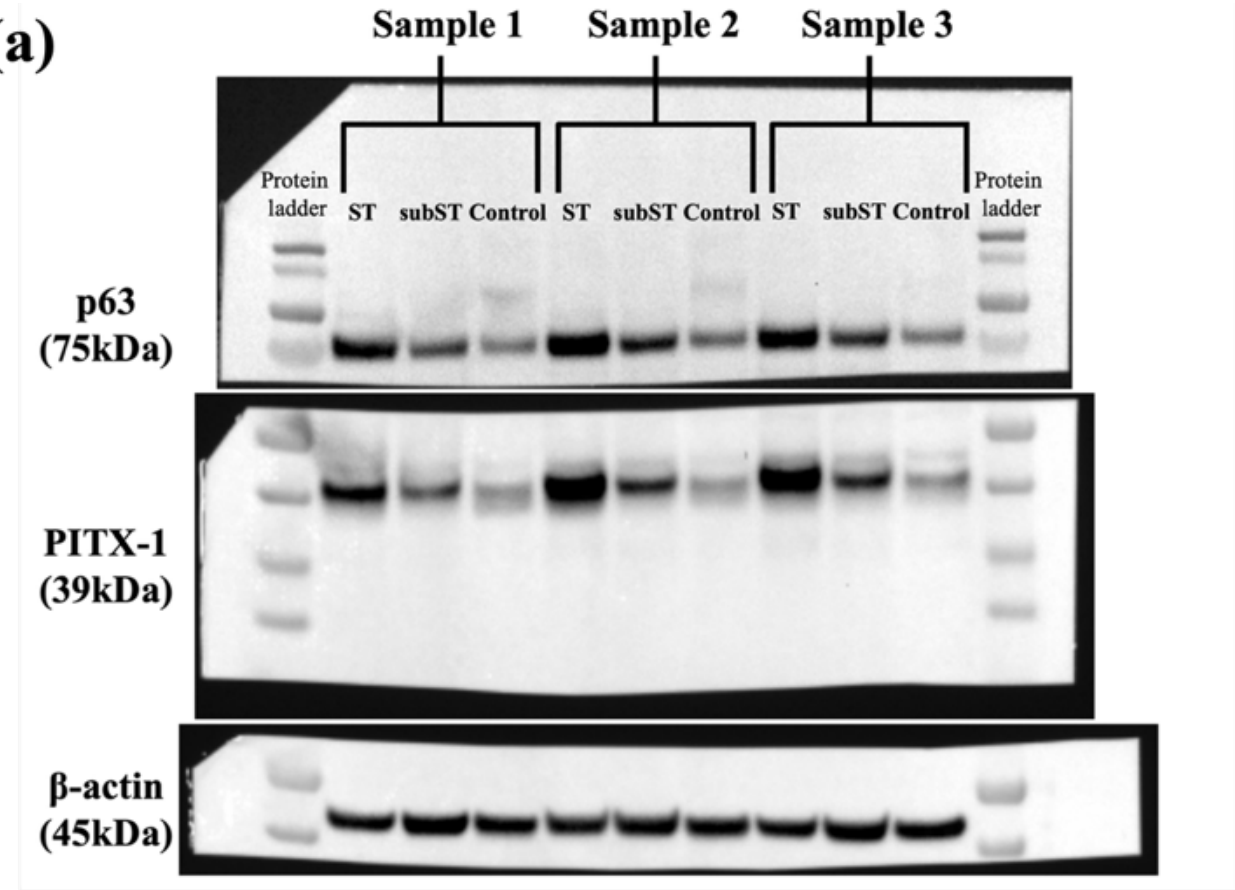

(b)

	BVW as p2			BVW p2			BLL p3		
	10% FBS	High Ca	Low Ca (control)	10% FBS	High Ca	Low Ca (control)	10% FBS	High Ca	Low Ca (control)
Densitometric quantification (P-63)	1,211,974	521,687	362,853	1,784,816	770,184	436,604	1,224,928	780,216	492,544
Normalized to β-actin (PITX-1)	5,232,532	1,795,653	1,643,895	6,788,798	2,662,357	1,374,489	7,793,831	3,031,004	1,844,458

Supplementary Figure S4

(a) Western blot image of p63 and PITX-1 of the passage 2 (p2) standard (ST) and sub-standard (subST) cultured oral mucosa epithelial cell sheets (COMECS)

As a control, cell lysates were obtained from p2 oral keratinocytes cultured in Low Ca^{2+}, serum free, EpiLife medium. β-actin was used as a loading control.

(b) Densitometric quantification of p63 and PITX-1 band intensities from three biological replicates, normalized to β-actin (p2 oral keratinocytes cultured in low-Ca2+, serum-free EpiLife medium).

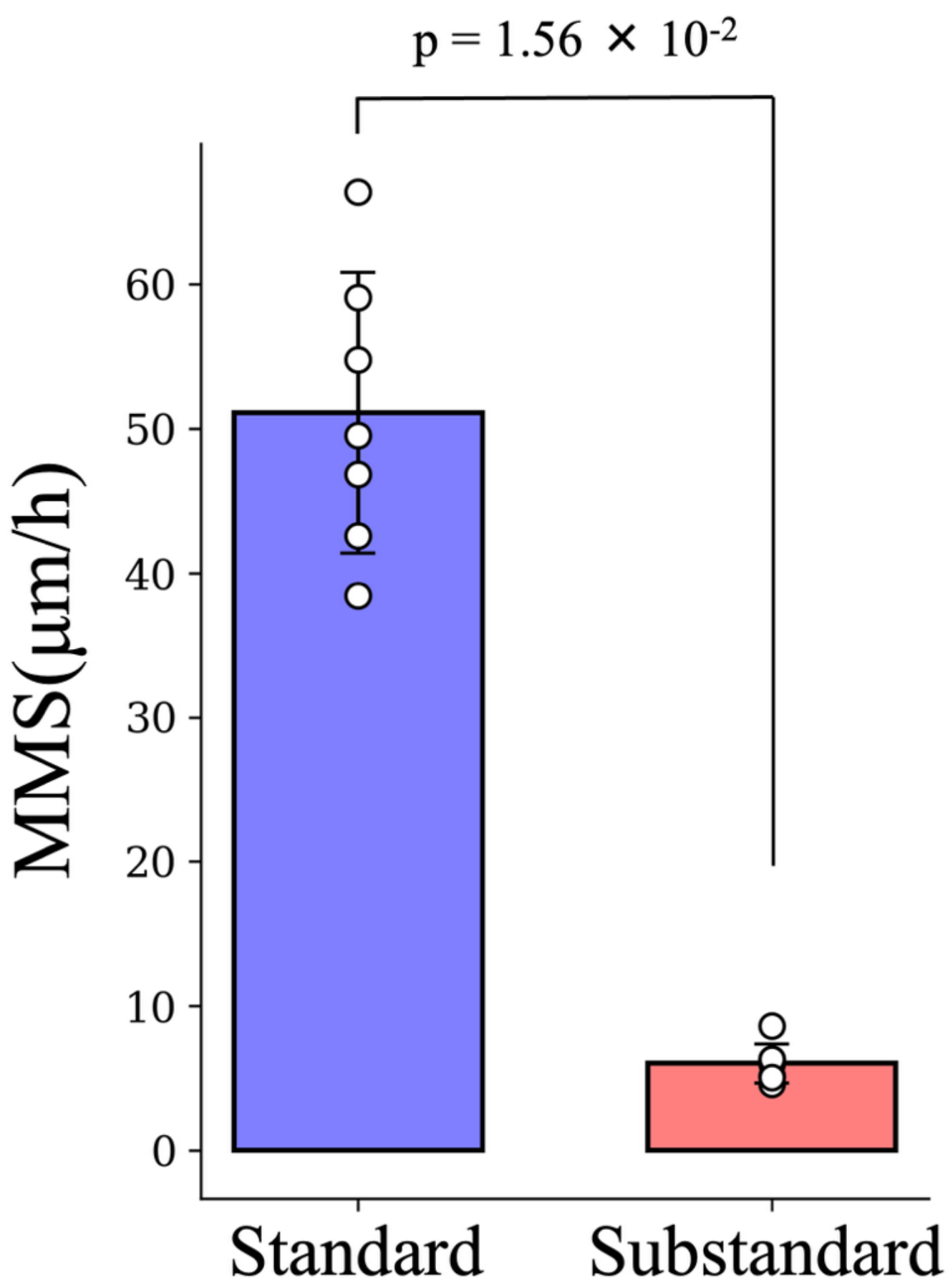

Supplementary Figure S5 Mean motion speed (MMS) of passage 2 (p2) standard and sub-standard COMECS after time-lapse microscopy

Each dot represents an individual measurement. Data are shown as mean ± standard deviation (SD). The p2 standard COMECS showed a higher MMS than p2 substandard COMECS ($p < 0.05$; Wilcoxon signed-rank test). MMS reflects the overall magnitude of cell movement, but does not assess the local directional synchrony that characterizes the undulating motion pattern of COMECS.
